# Microsomal Prostaglandin E Synthase-1 Facilitates an Intercellular Interaction between CD4^+^ T Cells through IL-1β Autocrine Function in Experimental Autoimmune Encephalomyelitis

**DOI:** 10.3390/ijms18122758

**Published:** 2017-12-19

**Authors:** Takako Takemiya, Chisen Takeuchi, Marumi Kawakami

**Affiliations:** 1 Medical Research Institute, Tokyo Women’s Medical University, Tokyo 162-8666, Japan; kawakami.marumi@twmu.ac.jp; 2 Department of Neurology, Tokyo Metropolitan Kita Medical and Rehabilitation Center for the Disabled, Tokyo 114-0033, Japan; chisentakeuchi@gmail.com

**Keywords:** interleukin-1β (IL-1β), prostaglandin E_2_ (PGE_2_), microsomal prostaglandin synthetase-1 (mPGES-1), *mPGES*-*1*-deficient (*mPGES-1^−/−^*) mice, CD4-positive T cells (CD4^+^ T cells), interleukin-17 (IL-17), experimental allergic encephalomyelitis (EAE), multiple sclerosis (MS), vascular endothelial cells (VECs), myelin oligodendrocyte glycoprotein_35–55_ peptide (MOG_35–55_)

## Abstract

Microsomal prostaglandin synthetase-1 (mPGES-1) is an inducible terminal enzyme that produces prostaglandin E_2_ (PGE_2_). In our previous study, we investigated the role of mPGES-1 in the inflammation and demyelination observed in experimental autoimmune encephalomyelitis (EAE), an animal model of multiple sclerosis, using *mPGES*-*1*-deficient (*mPGES-1^−/−^*) and wild-type (wt) mice. We found that mPGES-1 facilitated inflammation, demyelination, and paralysis and was induced in vascular endothelial cells and macrophages and microglia around inflammatory foci. Here, we investigated the role of interleukin-1β (IL-1β) in the intercellular mechanism stimulated by mPGES-1 in EAE spinal cords in the presence of inflammation. We found that the area invaded by CD4-positive (CD4^+^) T cells was extensive, and that PGE_2_ receptors EP1–4 were more induced in activated CD4^+^ T cells of wt mice than in those of *mPGES*-*1^−/−^* mice. Moreover, IL-1β and IL-1 receptor 1 (IL-1r1) were produced by 65% and 48% of CD4^+^ T cells in wt mice and by 44% and 27% of CD4^+^ T cells in *mPGES-1^−/−^* mice. Furthermore, interleukin-17 (IL-17) was released from the activated CD4^+^ T cells. Therefore, mPGES-1 stimulates an intercellular interaction between CD4^+^ T cells by upregulating the autocrine function of IL-1β in activated CD4^+^ T cells, which release IL-17 to facilitate axonal and myelin damage in EAE mice.

## 1. Introduction

In the brain, prostaglandin E_2_ (PGE_2_) is sequentially synthesized from arachidonic acid by cyclooxygenase-2 (COX-2) and microsomal PGE_2_ synthase (PGES)-1 (mPGES-1) during neuroinflammation. Moreover, mPGES-1 is induced in the vascular endothelial cells (VECs) of the brain to mediate endotoxin-induced fever [1] and neuronal loss elicited by kainic acid (KA) [2]. Multiple sclerosis (MS) is a central nervous system disease characterized by multifocal areas of leukocyte infiltration and demyelination. Inflammation is one of the most commonly observed pathological features of MS, and inflammatory mediators are known to modulate tissue damage in this disease. COX-2 was reported to be upregulated in microglia and macrophages in the brains of MS patients [3] and in brain VECs in experimental autoimmune encephalomyelitis (EAE), an animal model of MS [4]. In rodent EAE models using oligodendrocyte glycoprotein_35–55_ peptide (MOG_35–55_), histological examination revealed inflammatory foci with typical perivascular infiltration of mononuclear cells in the spinal cord and brain [5,6]. MOG_35–55_ EAE shows infiltration of the cerebral meninges at the lateral and the third ventricle and severe parenchymal infiltration in the spinal cord [6]. EAE disease is then manifested as an ascending flaccid paralysis with inflammation targeting the spinal cord [7,8]; accordingly, we focused on the inflammation of the spinal cord in this study.

In addition, PGE_2_ levels were previously shown to be increased in the spinal cords of EAE animals [9,10,11,12], while treatment with selective COX-2 inhibitors blocked the increase in spinal PGE_2_ and suppressed the development of paralysis in EAE [13,14,15]. The contribution of mPGES-1 to MS was revealed by showing that mPGES-1 induction in infiltrated macrophages facilitated the clinical progression of EAE mice [11,12]. We also demonstrated in *mPGES*-*1*-deficient (*mPGES-1^−/−^*) mice that PGE_2_, produced by mPGES-1 in VECs and macrophage and microglia around inflammatory foci, facilitated inflammation, demyelination, and paralysis in EAE [12]. Central PGE_2_ derived from endothelial and macrophage mPGES-1 may aggravate the spread of inflammation and local demyelination in the spinal cord; however, the mechanisms are unknown. In contrast, interleukin-1β (IL-1β) is known to aggravate EAE development and inflammation in the spinal cord. The administration of the recombinant interleukin-1 receptor antagonist (IL-1RN) to EAE rats delayed the onset of the disease and reduced its severity [16]. In addition, a defective interleukin-1 receptor 1 (IL-1r1) gene in mice has been associated with a complete resistance to EAE [17].

IL-1β is also generally known to facilitate PGE_2_ production. However, there is no evidence that IL-1β induction is conversely regulated by PGE_2_ or that IL-1β is related to the intercellular mechanism of EAE aggravation stimulated by mPGES-1. In addition, PGE_2_ is related to the differentiation of interleukin-17 (IL-17)-producing helper T cells (Th17), and it regulates the type 1 helper T cell (Th1)/Th17 immune responses of CD4-positive (CD4^+^) T cells. This regulation is accomplished via the EP2 and EP4 receptors, with both IL-23 and IL-1β [18,19]. PGE_2_ also enhances IL-17 expression through the EP4 receptor [20]. Moreover, Th17 differentiation is regulated by IL-1β signaling through IL-1r1 [21,22].

Therefore, we hypothesized that PGE_2_ synthesized by mPGES-1 regulates IL-1β autocrine function in CD4^+^ T cells and promotes intercellular communication between CD4^+^ T cells. Thus, we first investigated whether PGE_2_ synthesized by mPGES-1 could activate CD4^+^ T cells and EP receptors on CD4^+^ T cells. Next, we determined whether mPGES-1 could stimulate IL-1r1 appearance and the production of IL-1β and IL-17 in CD4^+^ T cells by assessing their cellular activity.

## 2. Results

### 2.1. mPGES-1 Induced in Endothelial Cells and Macrophages Aggravates EAE Paralysis

**Figure 1:**
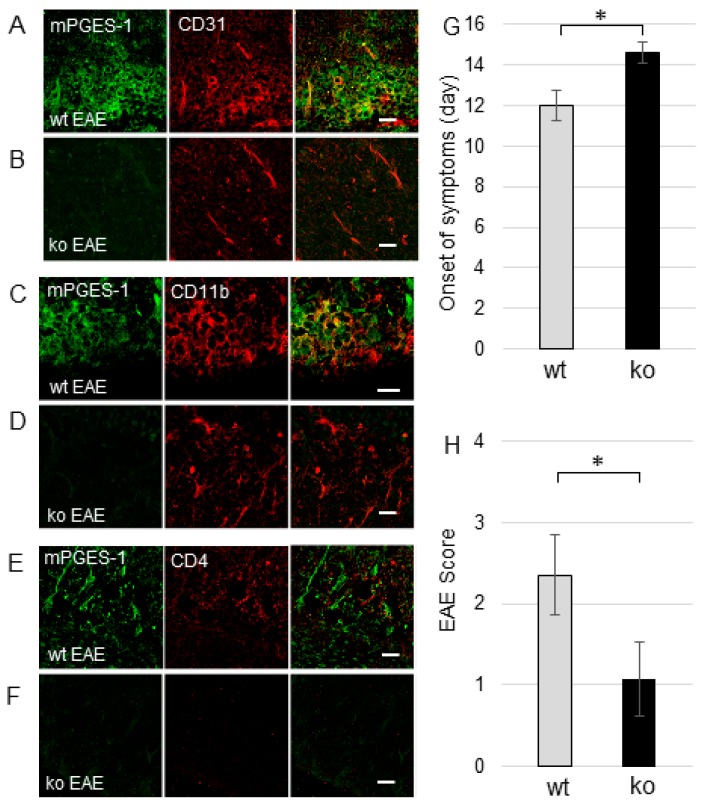
Expression of microsomal PGE_2_ synthase-1 (mPGES-1) and the effect of mPGES-1 on experimental autoimmune encephalomyelitis (EAE) paralysis. Immunohistochemistry image of mPGE-1 (green) with CD31 (**A**,**B**), CD11b (**C**,**D**), and CD4 (**E**,**F**) in the inflammatory region of spinal cords of wt EAE mice (**A**,**C**,**E**) and ko EAE (**B**,**D**,**F**) mice. Onset of symptoms (**G**) and the EAE score at day 19 (**H**) in EAE development. Scale bars (20 μm) for all images. wt, wild-type; ko, *mPGES-1^−/−^*. * *p* < 0.05.

In immunohistochemistry (IHC) experiments, mPGES-1 was expressed in CD31^+^ endothelial cells (ECs) and CD11b^+^ macrophages and microglia, but not in CD4^+^ T cells, which surrounded the other cells in the inflammatory region of the spinal cord in wild-type (wt) EAE mice (Figure 1A,C,E). In contrast, mPGES-1 was not expressed in *mPGES-1^−/−^* EAE mice (Figure 1B,D,F). Moreover, we did not detect CD19^+^ B cells in the inflammatory region (Figure S1). In the development of EAE, the onset of symptoms occurred significantly later in *mPGES-1^−/−^* EAE than in wt EAE (*p* = 0.0104, Figure 1G). In wt mice, the EAE score showed a gradual increase, with the score maintaining a high level, whereas the EAE score of *mPGES-1^−/−^* mice showed a temporary increase and then promptly decreased (Figure S2). Therefore, the EAE score at day 19 was significantly lower in *mPGES-1^−/−^* EAE than in wt EAE (*p* = 0.0403, Figure 1H). Thus, PGE_2_ produced by mPGES-1 facilitates EAE symptoms.

### 2.2. CD4^+^ T Cell Invasion in the EAE Spinal Cord is Facilitated by mPGES-1

**Figure 2:**
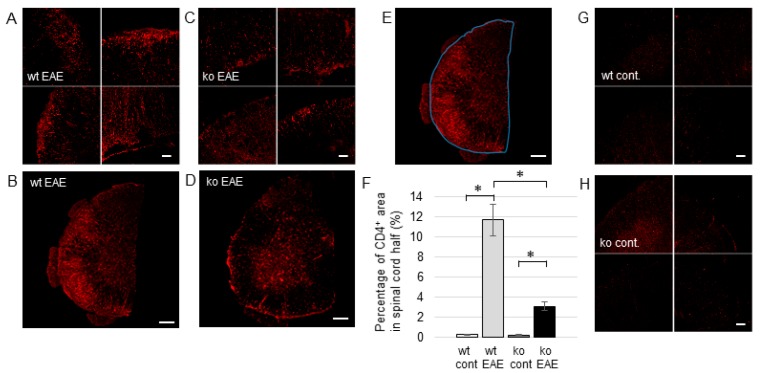
Distribution of CD4^+^ T cells in EAE spinal cords. Immunohistochemistry image showing CD4 in the inflammatory region of the spinal cord in wt EAE (**A**,**B**), ko EAE (**C**,**D**), wt control (**G**), and ko control (**H**) mice. Measurement of half size area in the spinal cord (**E**). Percentage of the CD4^+^ area in the EAE spinal cord half (**F**). Scale bar (20 μm) for (**A**,**C**,**G**,**H**), and (200 μm) for (**B**,**D**,**E**). wt, wild-type; ko, *mPGES-1^−/−^*; cont, naïve mice; EAE, EAE mice. * *p* < 0.0001.

CD4^+^ T cells formed a perivascular cluster and infiltrated into the parenchyma in wt EAE mice (Figure 2A,B); however, they were scattered around the vessels of the spinal cord in *mPGES-1^−/−^* EAE mice (Figure 2C,D). In addition, there were almost no CD4^+^ T cells in control spinal cords (Figure 2G,H). The percentage of the area invaded by CD4^+^ T cells was significantly higher in wt EAE (11.7 ± 1.6%) than in *mPGES-1^−/−^* EAE mice (3.1 ± 0.4%) (*p* < 0.0001, Figure 2E,F). This result suggests that PGE_2_ synthesized from mPGES-1 stimulates the activation of CD4^+^ T cells. We hypothesized that if PGE_2_ directly facilitates T cell activity, then the T cells must express the PGE_2_ receptor EP, and their IL-1β production may be increased. Therefore, we next examined the appearance of EP receptors and IL-1β in CD4^+^ T cells.

### 2.3. The Induction of EP Receptors and IL-1β in CD4^+^ T Cells is Regulated by mPGES-1

**Figure 3:**
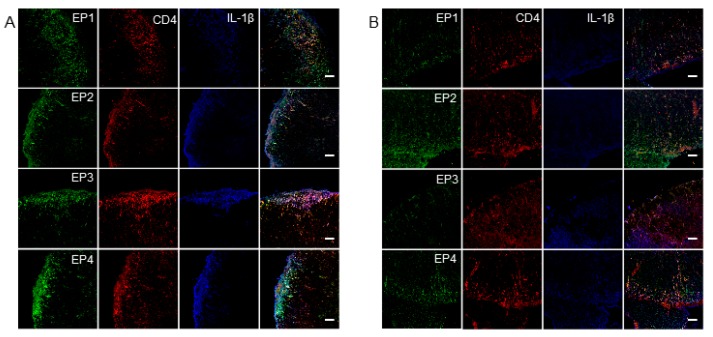
Expression of EP receptors and interleukin-1β (IL-1β) in CD4^+^ T cells in EAE spinal cords. Immunohistochemistry image showing EP1–4 (green), CD4 (red), and IL-1β (blue) in the inflammatory region of the spinal cords of EAE wild-type mice (**A**) and EAE *mPGES-1^−/−^* mice (**B**). Scale bar (50 μm) for all images.

**Figure 4:**
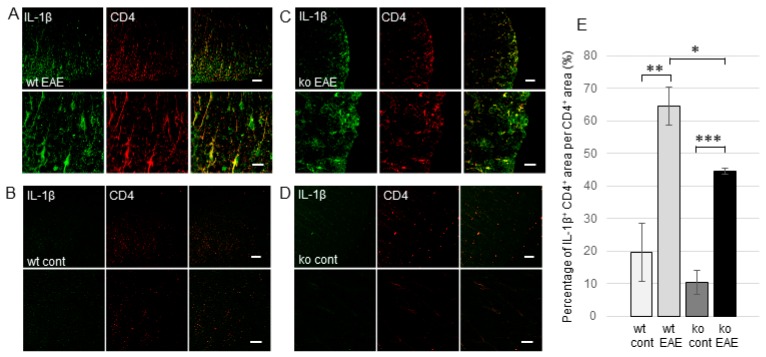
Expression of IL-1β in CD4^+^ T cells in the spinal cords of EAE and control mice. Immunohistochemistry image showing IL-1β (green) and CD4 (red) in the inflammatory region of the spinal cords of wt EAE (**A**), wt control (**B**), ko EAE (**C**), and ko control (**D**) mice. Percentage of the IL-1β^+^ CD4^+^ area in the CD4^+^ area of EAE spinal cords (**E**). Scale bar (50 μm) for upper panels and (20 μm) for lower panels of (**A**–**D**). wt, wild-type; ko, *mPGES-1^−/−^*; cont, naïve mice; EAE, EAE mice. * *p* < 0.05, ** *p* < 0.005, *** *p* < 0.0005.

**Figure 5:**
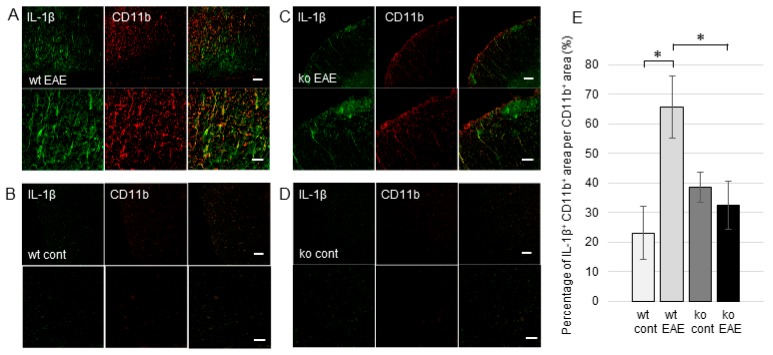
Expression of IL-1β in CD11b^+^ macrophages and microglia in the spinal cords of EAE and control mice. Immunohistochemistry image showing IL-1β (green) and CD11b (red) in the inflammatory region of the spinal cords of wt EAE (**A**), wt control (**B**), ko EAE (**C**), and ko control (**D**) mice. Percentage of the IL-1β^+^ CD11b^+^ area in the CD11b^+^ area of EAE spinal cords (**E**). Scale bar (50 μm) for upper panels and (20 μm) for lower panels of (**A**–**D**). wt, wild-type; ko, *mPGES-1^−/−^*; cont, naïve mice; EAE, EAE mice. * *p* < 0.05.

There was no expression of EP receptors or IL-1β in control spinal cords, regardless of the presence of mPGES-1 (Figure S3). EP receptors 1–4 were strongly stained and widely colocalized with CD4 in spinal cords in wt EAE mice, but only weakly stained and partially colocalized in *mPGES-1^−/−^* EAE mice (Figure 3 and Figure S4). In addition, IL-1β expression was observed in almost all CD4^+^ T cells in wt EAE mice, but in only a portion of CD4^+^ T cells in *mPGES-1^−/−^* EAE mice (Figure 3 and Figure S4). Because there were no major differences in the association with IL-1β between the different EP receptors, we examined IL-1β and CD4 double staining and compared it between wt and *mPGES-1^−/−^* EAE mice (Figure 4A–D). The percentage of the IL-1β^+^ CD4^+^ area per CD4^+^ area in the spinal cord was significantly higher in wt EAE mice (64.6 ± 5.8%) than in *mPGES-1^−/−^* EAE mice (44.5 ± 4.7%) (*p* = 0.0146, Figure 4E), suggesting that IL-1β production may be regulated by mPGES-1. In addition, IL-1β was also expressed in activated CD11b^+^ macrophages and microglia (Figure 5). The percentage of the IL-1β^+^ CD11b^+^ area per CD11b^+^ area in the spinal cord was significantly higher in wt EAE mice (65.7 ± 10.4%) than in *mPGES-1^−/−^* EAE mice (32.6 ± 8.1%) (*p* = 0.0128, Figure 5E), suggesting that IL-1β production in macrophages and microglia may also be regulated by mPGES-1.

### 2.4. IL-1r1 Expression in CD4^+^ T Cells is Controlled by mPGES-1

**Figure 6:**
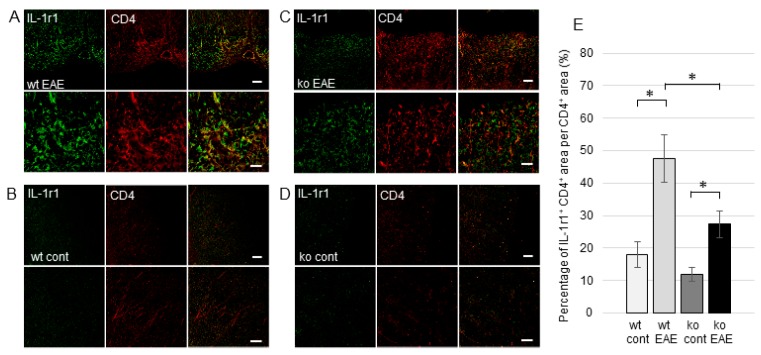
Expression of interleukin-1 receptor 1 (IL-1r1) in CD4^+^ T cells in the spinal cords of EAE and control mice. Immunohistochemistry image showing IL-1r1 (green) and CD4 (red) in the inflammatory region of the spinal cords of wt EAE (**A**), wt control (**B**), ko EAE (**C**), and ko control (**D**) mice. Percentage of the IL-1r1^+^ CD4^+^ area in the CD4^+^ area of EAE spinal cords (**E**). Scale bar (50 μm) for upper panels and (20 μm) for lower panels of (**A**–**D**). wt, wild-type; ko, *mPGES-1^−/−^*; cont, naïve mice; EAE, EAE mice. * *p* < 0.05.

There was no expression of IL-1r1 in control spinal cords regardless of the presence of mPGES-1 (Figure 6B,D). IL-1r1 staining was strong in the spinal cords of wt EAE mice (Figure 6A); however, it was rare in *mPGES-1^−/−^* EAE spinal cords (Figure 6C). Moreover, the percentage of the IL-1r1^+^ CD4^+^ area per CD4^+^ area was significantly higher in wt EAE mice (47.6 ± 7.4%) than in *mPGES-1^−/−^* EAE mice (27.3 ± 4.1%) (*p* = 0.0366, Figure 6E).

### 2.5. IL-17 Expression in CD4^+^ T Cells is Regulated by mPGES-1

**Figure 7:**
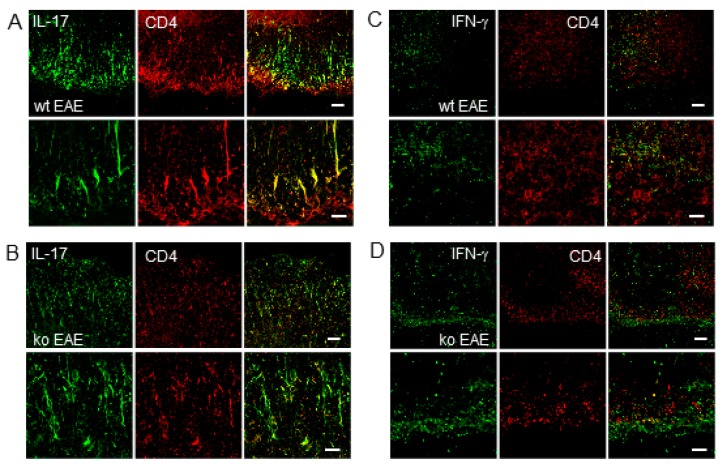
Expression of interleukin-17 (IL-17) and interferon-γ (IFN-γ) in CD4^+^ T cells in the spinal cords of EAE mice. Immunohistochemistry image showing IL-17 (green) and CD4 (red) in the inflammatory region of the spinal cords of wt EAE (**A**) and ko EAE (**B**) mice; and IFN-γ (green) and CD4 (red) in the inflammatory region of the spinal cords of wt EAE (**C**) and ko EAE (**D**) mice. Scale bar (50 μm) for upper panels and (20 μm) for lower panels of A–D. wt, wild-type; ko, *mPGES-1^−/−^*.

IL-17 was clearly expressed in activated CD4^+^ T cells with morphological changes in the spinal cords of wt EAE mice (Figure 7A), whereas the changes in T cells were limited and IL-17 staining was faint in *mPGES-1^−/−^* EAE spinal cords (Figure 7B). In contrast, interferon-γ (IFN-γ) staining was partially colocalized with CD4^+^ T cells in both wt and *mPGES-1^−/−^* EAE mice (Figure 7C,D).

## 3. Discussion

First, we discuss the role of PGE_2_, COX-2, and mPGES-1 in MS and EAE. In clinical research using leukocyte cultures from patients with definite MS, patients had higher baseline PGE expression than control subjects [23]. In addition, PGE_2_ expression is higher in peripheral blood monocytes from patients with chronic progressive MS [24], suggesting that PGE_2_ is related to MS development. PGE_2_ is sequentially synthesized from arachidonic acid by COX and PGES, and COX exists as two isoforms. COX-1 is constitutively active, while COX-2 is inducible. In MS patients, COX-2 is induced in chronic active lesions, specifically near damaged oligodendrocytes in macrophages and microglia [3]. COX-2 has also been reported to be localized to von-Willebrand factor-marked ECs in the spinal cord in EAE, especially 14 to 25 days after immunization [4], and it appears in CD11b^+^ cells 16 days after immunization in EAE [25].

Moreover, PGES has three isoforms: mPGES-1 [26], mPGES-2 [27], and cytosolic PGE_2_ synthase (cPGES) [28]. mPGES-2 and cPGES are constitutively expressed, while mPGES-1 is induced in macrophages or osteoblasts following proinflammatory stimulation and is functionally coupled with COX-2 [29]. In the brain, mPGES-1 is induced in vascular endothelial cells in fever [1] and KA-induced neuronal injury [2]. In EAE, mPGES-1 appears in F4/80^+^ or CD68^+^ macrophages in the spinal cord [11] and in CD31^+^ ECs and CD11^+^ macrophages and microglia in the spinal cord 19 days after immunization [12]. In the present study, we confirmed that mPGES-1 was expressed in CD31^+^ ECs (Figure 1A) and CD11^+^ macrophages and microglia at day 19 after immunization (Figure 1C) but not in CD4^+^ T cells (Figure 1E). In addition, the onset of symptoms occurred significantly later, and the EAE score at day 19 was significantly lower in *mPGES-1^−/−^* EAE mice compared to wt EAE mice (Figure 1G,H). The examination of EAE scores showed that *mPGES-1^−/−^* mice show significant impairment in the development of EAE for 26 days [11] or 35 days [12]. In the present study, the EAE score was only examined until day 19 (Figure S2); therefore, there was no significant difference in EAE scores between wt and *mPGES-1^−/−^* mice as shown by ANOVA. In our previous study, we monitored the EAE score for 28 days and found significant differences after day 19. Furthermore, *mPGES-1^−/−^* mice exhibited a decrease in the activation of microglia of the spinal cord related to neuropathic pain [30]. These findings suggest that inducible PGE_2_, synthesized by COX-2 and mPGES-1 in macrophages and microglia and VECs, stimulates pathological changes in the spinal cord in EAE and leads to EAE paralysis.

We next discuss the role of IL-1β in MS and EAE. In the clinic, significantly higher values of IL-1β have been detected in the cerebrospinal fluid and serum of MS patients [31]. Several studies have provided evidence that IL-1β plays a crucial role in EAE. First, the administration of recombinant IL-1RN to EAE rats resulted in a delay in the onset of the disease and reduced its severity [16]. In addition, a defective IL-1r1 gene in mice was associated with a complete resistance to EAE [17]. Recently, IL-1β was reported to be the main protagonist of EAE, whereas IL-1α is dispensable [32].

IL-1β is known to stimulate the induction of COX-2. Intraperitoneal injection of IL-1β induces the expression of COX-2 mRNA in VECs in the brain [33] and leads to the induction of COX-2 mRNA in cultured primary hippocampal neurons [34]. Moreover, the main inducer of central COX-2 upregulation in the spinal cord is IL-1β, which thus promotes the production of PGE_2_ and contributes to inflammatory pain hypersensitivity [35]. COX-2 inhibitors prevent the IL-1β-induced increase in PGE_2_ production in the brain [36], suggesting that IL-1β regulates PGE_2_ production through the synthesis of COX-2. In addition, cross-talk between tumor cells and microvascular ECs upregulate COX-2 and mPGES-1, which are both strongly inhibited by an IL-1-receptor antagonist [37]. Thus, IL-1β plays an important role in the induction of COX-2 and mPGES-1 to produce pathophysiological PGE_2_, which promotes inflammation. These data suggest that IL-1β may stimulate the induction of COX-2 and mPGES-1 in VECs or macrophages and microglia in the spinal cord in EAE. In contrast, there is no evidence that IL-1β is controlled by PGE_2_ in EAE spinal cords.

CD4^+^ T cells formed a perivascular cluster and infiltrated the parenchyma in the spinal cords of wt EAE mice but were scattered around the vessel in the spinal cords in *mPGES-1^−/−^* EAE mice (Figure 2A–D) [12]. Since there were no T cells in the spinal cords of normal control wt or *mPGES-1^−/−^* mice (Figure 2G,H), CD4^+^ T cells infiltrated the spinal cord through the disrupted blood–spinal barrier in EAE. The percentage of the CD4^+^-stained area in half of the spinal cord of wt EAE mice was significantly higher than that in *mPGES-1^−/−^* EAE mice (Figure 2E,F). Thus, mPGES-1 might regulate the infiltration or the activation of CD4^+^ T cells (or both) in EAE. If PGE_2_ directly activates CD4^+^ T cells, T cells must express the PGE_2_ receptor EP, and IL-1β expression may be further upregulated by PGE_2_ in wt EAE compared with *mPGES-1^−/−^* EAE mice.

We first examined EP expression in CD4^+^ T cells. There was a low EP expression in normal control wt mice or *mPGES-1^−/−^* mice (Figure S2). In contrast, EP receptors 1–4 were strongly stained and widely colocalized with CD4 in the spinal cords of wt EAE mice, but were weakly stained and only partially colocalized with CD4 in *mPGES-1^−/−^* EAE mice (Figure 3 and Figure S4). Because all four EP receptors were shown to be expressed in neurons and induced in VECs under conditions of hypoxic–ischemic encephalopathy [38], EP receptors are known to be inducible in pathological conditions. In previous studies, T cells were regulated by PGE_2_, and this effect was mediated by EP2 or EP4 [39,40,41]. Moreover, IL-1β upregulated EP2 and EP4 in primary cultured hippocampal neurons [42]. Furthermore, we found that EP^+^ CD4^+^ T cells were almost entirely colocalized with IL-1β in wt EAE mice, but they rarely expressed IL-1β in the *mPGES-1^−/−^* EAE mice in the current study (Figure 3 and Figure S3). CD4^+^ T cells might be stimulated by PGE_2_ produced by mPGES-1, through EP receptors, to increase IL-1β production in EAE; however, we could not determine which specific EP receptor was crucial for producing IL-1β in CD4^+^ T cells using IHC because individual T cells may express multiple EP receptor types.

Second, we investigated IL-1β expression in CD4^+^ T cells in wt EAE and *mPGES-1^−/−^* EAE mice. There was no co-expression of IL-1β and CD4 in normal control wt mice or *mPGES-1^−/−^* mice (Figure 4B,D). In contrast, we found IL-1β expression in almost all CD4^+^ T cells in wt EAE (Figure 4A) mice, but in only a few CD4^+^ T cells in *mPGES-1^−/−^* EAE mice (Figure 4C). The percentage of the IL-1β^+^ CD4^+^ area per CD4^+^ area in the spinal cords of wt EAE mice was significantly higher than that in *mPGES-1^−/−^* EAE mice (Figure 4E). During in vitro Th17 differentiation, pro-IL-1β expression is strongly induced, suggesting that CD4^+^ T cells are a major cellular source of IL-1β during EAE pathogenesis [43]. Previous reports have shown that IL-1β is highly expressed in infiltrating macrophages [44], and IL-1β mRNA is induced in peritoneal leukocytes right after the initial EAE induction [45]. Moreover, activated macrophages are considered to be the predominant source of IL-1β in the brain [46]. We also observed IL-1β expression in CD11b^+^ macrophages in spinal cords (Figure 5). In wt EAE mice, IL-1β production in CD11^+^ macrophages and microglia was significantly increased compared with that of *mPGES-1^−/−^* EAE mice (Figure 5). However, we recently determined that the contribution of macrophages to the total production of IL-1β is minor in EAE pathogenesis [32,44]. The IL-1β released from macrophages and microglia might induce mPGES-1 in other macrophages and microglia and play roles in the inflammatory loop of the chronic phase of EAE [47]. In addition, the release of IL-1β from astrocytes might regulate the permeability of the blood–spinal barrier and the infiltration of immune cells into the spinal cord [48].

We next studied the expression of IL-1r1 in CD4^+^ T cells in the spinal cord. There was no co-expression of IL-1r1 and CD4 in normal control wt mice or *mPGES-1^−/−^* mice (Figure 6B,D). In contrast, IL-1r1 was expressed in almost all CD4^+^ T cells in wt EAE mice (Figure 6A). Since IL-1r1 was detected on Th17 but not on TH1 cells in EAE mice [43], and because early Th17 differentiation is regulated by IL-1β signaling through IL-1r1 [21,22], many of the CD4^+^ T cells had the same properties as Th17 cells in the wt EAE mice used in the present study. Interestingly, IL-1r1 was rarely expressed in CD4^+^ T cells in *mPGES-1^−/−^* EAE mice (Figure 6C). The percentage of the IL-1r1^+^CD4^+^ area per CD4^+^ area of the spinal cord in wt EAE was significantly higher than that in *mPGES-1^−/−^* EAE (Figure 6E). This result suggests that mPGES-1 stimulates IL-1r1 expression in CD4^+^ T cells and promotes their differentiation to Th17 by IL-1β. Therefore, mPGES-1 modulates the IL-1β-dependent autocrine regulation of CD4^+^ T cells with TH17 properties.

Finally, we discuss the discrepancy between the appearance of IL-1β and IL-1r1 in CD4^+^ T cells. In this study, we investigated their expression using spinal cords obtained 19 days after immunization. We previously found a significant difference between wt EAE and *mPGES-1^−/−^* EAE in their inflammation and demyelination at 19 and 35 days after immunization, however there was a remarkable histological change at day 35 [12]. Therefore, the balance of IL-1β and IL-1r1 production may be altered during EAE development. Moreover, IL-1β is known to regulate blood–brain barrier (BBB) permeability [49,50], and IL-1r1 signaling controls chemokine CXCL12 expression at the BBB, affecting the severity of EAE [51]. Thus, endothelial IL-1r1 is an important receptor in EAE pathology [32,52]. Furthermore, IL-1r1 is essential for the efficient activation of microglia in response to brain injury [53]. Therefore, IL-1β produced in CD4^+^ T cells might affect not only CD4^+^ T cells but also ECs or macrophages and microglia in the inflammatory region.

In experiments to investigate the production of IL-17 or IFN-γ in CD4^+^ T cells in the EAE spinal cord, IL-17 staining was clearly colocalized with many morphologically changed CD4^+^ T cells in wt EAE mice (Figure 7A); however, the change in T cell morphology was limited, and IL-17 staining was faint in *mPGES-1^−/−^* EAE mice (Figure 7B). In contrast, there were few CD4^+^ T cells that co-expressed IFN-γ with CD4^+^ T cells in either wt (Figure 7C) or *mPGES-1^−/−^* EAE mice (Figure 7D). The analysis of Th1 and Th17 cells in culture supernatants showed that IFN-γ expression is higher in Th1 cells, and IL-17 expression is higher in Th17 cells [54]. Our results suggest that mPGES-1 facilitates the differentiation of CD4^+^ T cells toTh17 cells. The CD4^+^ T cell characteristics are altered according to the timing of the secretion of IL-17, IFN-γ, or both, after immunization in the spinal cord in EAE. Initially, the number of CD4^+^ IFN-γ^+^ T cells is increased, as is that of CD4^+^ IL-17^+^ T cells, though more gradually [55]. When EAE mice are treated with anti-IL-17 antibodies before the increase in CD4^+^ IL-17^+^ T cells, the EAE incidence and EAE score are reduced, suggesting that CD4^+^ T cells regulate tissue inflammation by producing IL-17 [56]. In our previous study, histopathological examination revealed significant differences between wt and *mPGES-1^−/−^* mice in the inflammation and demyelination of their spinal cords at days 19 and 35 after EAE immunization [12]. We confirmed this result at day 19 in the present study (data not shown). PGE_2_ is reportedly related to Th17 differentiation. PGE_2_ and IL-23 plus IL-1β differentially regulate the Th1/Th17 immune response of CD4^+^ T cells via the EP2 and EP4 receptors [18,19] and enhance IL-17 expression through EP4 [20]. The main source of PGE_2_ might be ECs and macrophages, and PGE_2_ might be primarily synthesized by mPGES-1. We did not detect CD19^+^ B cells in the inflammatory regions of EAE spinal cords. In rat and marmoset models of MOG-induced EAE, demyelination is antibody-dependent and reproduces the immunopathology seen in many MS patients, but, in mice, inflammation of the spinal cord can result in demyelination in the absence of a MOG-specific B cell response [57]. In addition, EAE development in B cell-deficient mice was shown to be the same as in wt mice [58]. Therefore, B cells may not be necessary for primary demyelination in MOG-induced EAE in mice.

**Scheme 1:**
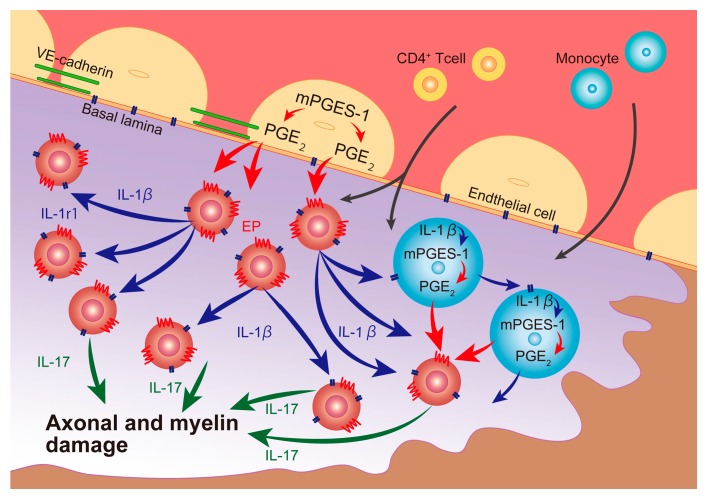
PGE_2_ synthesized by mPGES-1 facilitates the invasion of CD4^+^ T cells by inducing EP and IL-1r1 receptors, and potentiates IL-1β production, which exerts an autocrine signaling through IL-1r1 in wt EAE mice. This intercellular interaction affects IL-17 production, which finally aggravates inflammation and demyelination. Red arrows show production and secretion of PGE_2_, blue arrows show IL-1β, green arrows show IL-17, and gray arrows show cells infiltration from blood to spinal cord.

**Scheme 2:**
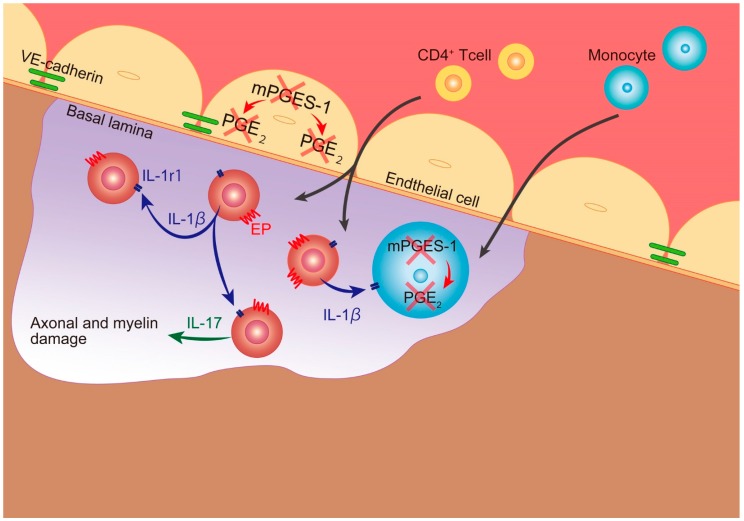
In contrast, in *mPGES-1^−/−^* mice, CD4^+^ T cell invasion is suppressed along with EP receptor appearance, and, subsequently, IL-1r1 appearance and the production of IL-1β and IL-17 are reduced. Red arrows show production and secretion of PGE_2_, blue arrows show IL-1β, green arrows show IL-17, and gray arrows show cells infiltration from blood to spinal cord.

In this study, we demonstrated that the invasion of CD4^+^ T cells was significantly increased by mPGES-1-derived PGE_2_ through the induction of EP and IL-1r1 receptors on CD4^+^ T cells and the potentiation of IL-1β production. IL-β1 then exerted an autocrine signaling through IL-1r1 (Scheme 1). These intercellular interactions affected the production of IL-17, which ultimately aggravated inflammation and demyelination (Scheme 1). This signaling pathway is regulated by mPGES-1; therefore, mPGES-1-deficient mice showed a suppression of CD4^+^ T cell invasion with a decrease in IL-1β production, EP and IL-1r1 receptor expression, and IL-17 production (Scheme 2).

## 4. Materials and Methods

### 4.1. Mice

Seven female *mPGES-1^−/−^* mice, 8 weeks of age, and seven female age-matched C57BL/6J mice were used in this study. The *mPGES-1^−/−^* mice were generated as previously described [59]. We used untreated female wt mice and *mPGES-1^−/−^* mice as a normal control. Seven mice were included in each group. The mice were housed four or five per cage in a room maintained at 24 ± 2 °C in a standard 12 h light-dark cycle and had access to standard chow and water ad libitum. All animal experiments were approved by the Ethical Review Committee of Animal Experiments and Gene Recombination Experiment Safety Committee of Tokyo Women’s Medical University (14-91/14-44, 29 March 2014).

### 4.2. Induction and Assessment of EAE

Mice were immunized by subcutaneous injection with 250 μg myelin oligodendrocyte glycoprotein_35–55_ peptide (MOG_35–55_, MEVGWYRSPFSRVVHLYRNGK, purity > 95%, Operon Technology, Tokyo, Japan) in complete Freund’s adjuvant (Difco, Detroit, MI, USA). The mice also received two intraperitoneal injections of 500 ng of pertussis toxin each (Seikagaku Corporation, Tokyo, Japan), once on the day of immunization and again two days later. The mice were observed daily, and we assessed the progression of EAE using the following scoring system: (0) no detectable signs of paralysis; (1) completely limp tail; (2) loss of the righting reflex; (3) partial hind limb paralysis; (4) complete hind limb paralysis; (5) total paralysis of all four limbs; and (6) death. Mice that were scored as 5 for two consecutive days were immediately euthanized.

### 4.3. Immunohistochemistry

For immunohistochemical detection, the mice were sacrificed under deep anesthesia induced by an intraperitoneal injection of pentobarbital (120 mg/kg) 19 days after immunization. The spinal cords were quickly removed and frozen with a dry ice/acetone mixture. Sections were cut at a thickness of 10 μm and left to air dry at room temperature for 30 min, after which they were rinsed with PBS. Next, the sections were treated with 10% normal goat serum for 5 h and then incubated with antibodies. The sections were incubated with anti-mPGES-1 antibodies (Cayman Chemical, Ann Arbor, MI, USA, #160140, 1:250 dilution) overnight and then with anti-EP1 (Bioss, Woburn, MA, USA, bs-6316R, 1:100), anti-EP2 (Bioss, Woburn, MA, USA, bs-4196R, 1:100), anti-EP3 (Santa Cruz Biotechnology, Inc., Dallas, TX, USA, sc-20676, 1:100), anti-EP4 (Bioss, Woburn, MA, USA, bs-8538R, 1:100), anti-IL-1β (Santa Cruz Biotechnology, Inc., Dallas, TX, USA, sc-15325, 1:100, Cloud-Clone, Houston, TX, USA, MAA563Mu21, 1:25), or anti-IL-1r1 (Santa Cruz Biotechnology, Inc., Dallas, TX, USA, sc-689, 1:100) antibodies for two nights at 4 °C with slow shaking. To visualize the ECs, macrophages and microglia, CD4-positive T lymphocytes, and CD19-positive B lymphocytes, some of the sections were double stained with anti-CD31 (BD Bioscience, La Jolla, CA, USA, #550274, 1:20), anti-CD11b (BD Bioscience, La Jolla, CA, USA, #550282, 1:25), anti-CD4 (BD Bioscience, La Jolla, CA, USA, #550278, 1:50), or anti-CD19 (Thermo Fisher, Waltham, MA, USA, #14-0194-82, 1:100; Bioss, Woburn, MA, USA, bs-0079R, 1:100) antibodies. After removal of the primary antibody, the sections were incubated with FITC-labeled anti-rabbit IgG (1:150) to label mPGES-1, EP1–4, IL-1β, and IL-1r1, or with Cy3-labeled anti-rat IgG (1:150) to label CD4, CD31, CD11b, or CD19, for 5 h at room temperature with slow shaking. In addition, we examined the production of cytokines, such as IL-17 or interferon-γ (IFN-γ), in CD4-positive T lymphocytes. The spinal cord sections were treated with 10% normal donkey serum for 5 h and then incubated with anti-CD4 antibodies (Thermo Fisher, Waltham, MA, USA, #AF554, 1:25) and either anti-IL-17 (Thermo Fisher, Waltham, MA, USA, USA, #17-7177-81, 1:100) or anti-IFN-γ (Thermo Fisher, Waltham, MA, USA, #MM700, 1:100) antibodies 3 overnight at 4 °C with slow shaking. After removal of the primary antibody, the sections were incubated with FITC-labeled anti-rat IgG (1:150) to label IL-17 and IFN-γ, or with Cy3-labeled anti-goat IgG (1:150) to label CD4, for 5 h at room temperature with slow shaking. Fluorescent images were obtained using a confocal microscope (Carl Zeiss LSM510 and LSM710, Oberkochen, Germany).

### 4.4. Image Analysis

To analyze the fluorescent images, we measured the CD4-stained area in the inflammatory half of the spinal cord using Carl Zeiss software ZEN2.3, and then calculated the ratio of the CD4-stained area to the total half-spine area. In addition, we measured the IL-1β- or IL-1r1-stained area co-localized with CD4 in each experiment and calculated the ratio of the costained area to the CD4-stained area using the colocalization function of the Carl Zeiss software AIM (Carl Zeiss LSM510 and LSM710, Oberkochen, Germany).

### 4.5. Statistical Analysis

The data are presented as the means ± standard error (s.e.). The statistical analysis was performed using independent Student’s *t*-tests with assumption of equal variance, and significance was determined at a *p*-value < 0.05.

## 5. Conclusions

We determined that PGE_2_, synthesized by mPGES-1 in ECs and macrophages and microglia, facilitates an intercellular interaction between CD4^+^ T cells by regulating IL-1β autocrine function in EAE development.

## Supplementary Materials

Supplementary materials can be found at www.mdpi.com/1422-0067/18/12/2758/s1.

## Abbreviations

PGE_2_	Prostaglandin E_2_
COX-2	Cyclooxygenase-2
PGES	PGE_2_ synthase
mPGES-1	Microsomal prostaglandin synthetase-1
VECs	Vascular endothelial cells
KA	Kainic acid
MS	Multiple sclerosis
EAE	Experimental allergic encephalomyelitis
MOG_35–55_	Oligodendrocyte glycoprotein_35–55_ peptide
*mPGES-1^−/−^*	*Microsomal prostaglandin synthetase-1*-deficient
IL-1β	Interleukin-1β
IL-1RN	Interleukin-1 receptor antagonist
IL-1r1	Interleukin-1 receptor 1
IL-17	Interleukin-17
Th17	Interleukin-17-producing helper T cell
Th1	Type 1 helper T cell
CD4^+^ T cells	CD4-positive T cells
IHC	Immunohistochemistry
wt	Wild-type
IFN-γ	Interferon-γ
cPGES	Cytosolic PGE_2_ synthase
BBB	Blood-brain barrier
