# Supplementary material for: Microsomal Prostaglandin E Synthase-1 Facilitates an Intercellular Interaction between CD4+ T Cells through IL-1β Autocrine Function in Experimental Autoimmune Encephalomyelitis
Source: Int J Mol Sci. 2017 Dec 19;18(12):2758. doi: 10.3390/ijms18122758 (PMC5751357; doi:10.3390/ijms18122758)
Supplement: Supplementary file 1 [file ijms-18-02758-s001.pdf]

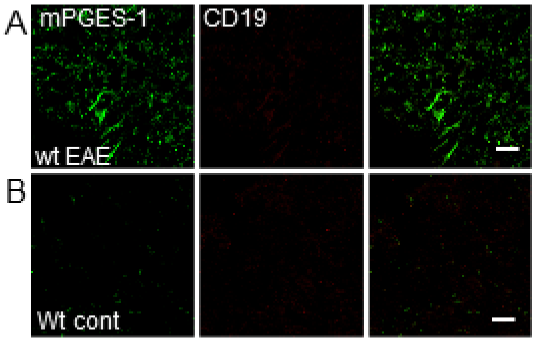

Supplementary Figure 1.
Immunohistochemistry image of microsomal PGE_2 synthase-1 (mPGES -1) (green) with CD19 (red) in the inflammatory region of spinal cords of EAE wild-type (wt) mice (**A**) and control wt mice (**B**). Scale bars (20 μm) for all images.

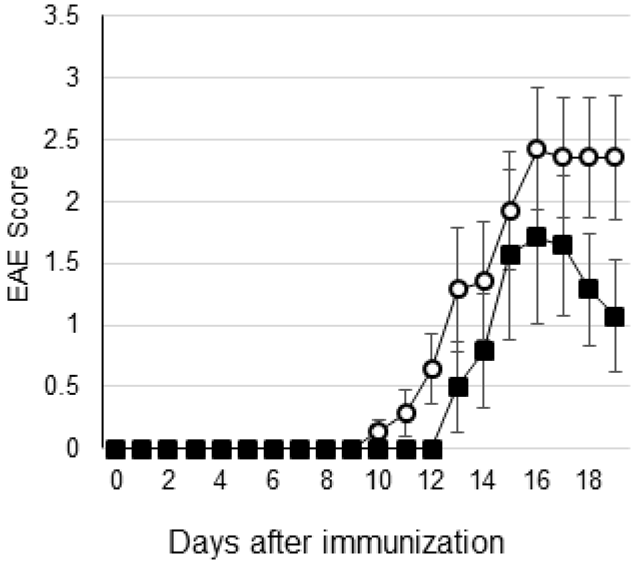

Supplementary Figure 2.

EAE development in wt and *mPGES-1*-deficient (*mPGES-1*$^{-/-}$) mice. The EAE score gradually increased and maintained a high level in wt mice (open circle) but promptly decreased in *mPGES-1*$^{-/-}$ mice (closed square).

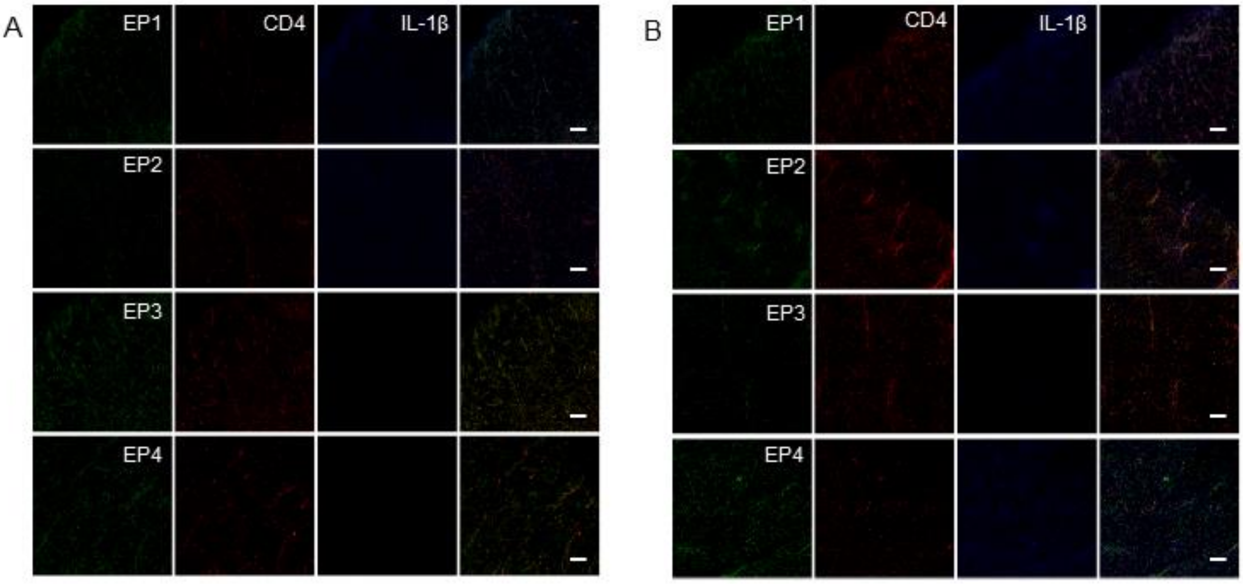

Supplementary Figure 3.

Expression of EP receptors and IL-1β in $CD4^+$ T cells in the control spinal cord. Immunohistochemistry image showing EP 1-4 (green), CD4 (red) and IL-1β (blue) in the spinal cords of control wt mice (A) and control *mPGES-1*$^{-/-}$ mice (B). Scale bar (50 μm) for all images.

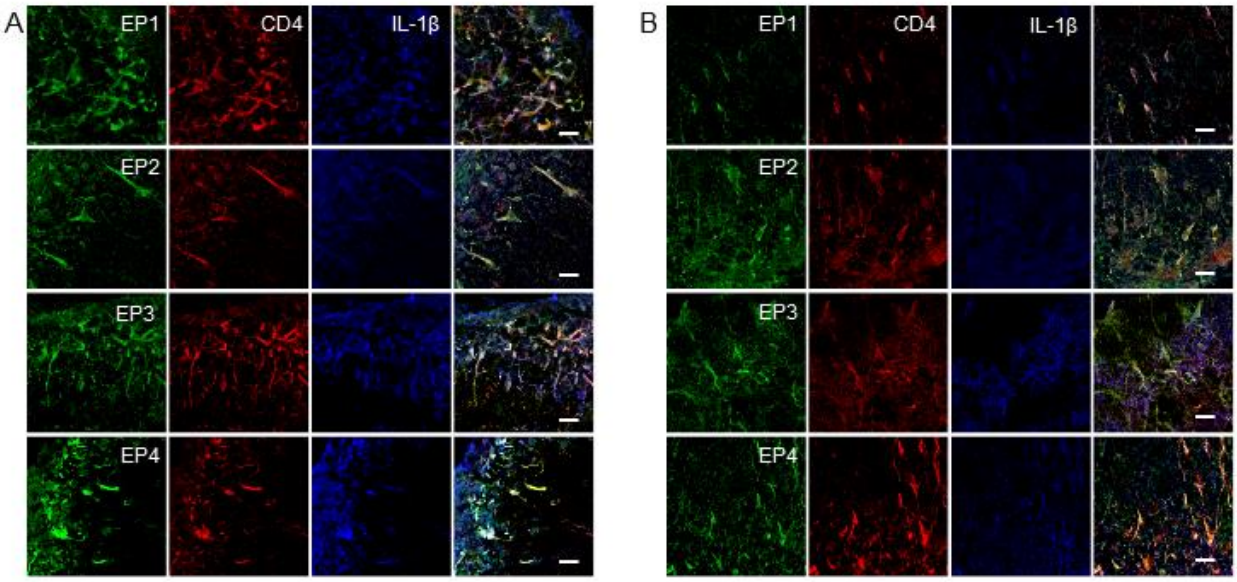

Supplementary Figure 4.

Expression of EP receptors and IL-1β in $CD4^+$ T cells in the EAE spinal cord. Immunohistochemistry image showing EP 1-4 (green), CD4 (red) and IL-1β (blue) in the inflammatory region of spinal cords of EAE wt mice (A) and EAE *mPGES-1*$^{-/-}$ mice (B). Scale bar (20 μm) for all images.
